# Estrogen to progesterone ratio is associated with conceptus attachment in dairy cows receiving artificial insemination after Double-Ovsynch but not estrus^†^

**DOI:** 10.1093/biolre/ioae102

**Published:** 2024-06-25

**Authors:** Thainá Minela, Alisson Santos, J Richard Pursley

**Affiliations:** Department of Animal Science, Michigan State University, East Lansing, USA; Department of Animal Science, Michigan State University, East Lansing, USA; Department of Animal Science, Michigan State University, East Lansing, USA

**Keywords:** PSPB, steroid hormone, conceptus attachment, pregnancy loss

## Abstract

Prediction of pregnancy survival in lactating dairy cows can be determined by the conceptus attachment timeframe via daily pregnancy-specific protein B (PSPB) monitoring. All factors contributing to reduced fertility in dairy cows receiving AI following estrus detection remain unclear. This study aimed to determine differences in time to conceptus attachment in lactating cows treated with the fertility program Double-Ovsynch compared to cows that were detected in estrus. Additionally, we investigated various pre- and post-conception factors potentially influencing fertility outcomes. We hypothesized that AI following a natural estrus detected with automated activity monitors would lead to an extended time to conceptus attachment and lower PSPB concentrations post-attachment compared to Double-Ovsynch. There were no differences in the average time to conceptus attachments between treatments. However, cows inseminated post-estrus that experienced pregnancy loss between conceptus attachment and 60–66 days post-AI exhibited diminished PSPB concentrations on Days 2 and 3 following conceptus attachment. Steroid hormone interactions were assessed with radioimmunoassay to determine the ratios of estrogen to progesterone concentrations on the day of the luteinizing hormone (LH) surge. Notably, estrogen to progesterone ratio proved to predict conceptus attachment in cows subjected to Double-Ovsynch but not in those inseminated post-estrus detection surge. In conclusion, the estrogen to progesterone ratio measured around the time of the pre-ovulatory LH surge emerges as a potentially effective tool for estimating the fertility potential of lactating dairy cows undergoing timed AI, particularly in the context of the Double-Ovsynch program.

## Teaser

Controlled hormonal intervention of ovarian dynamics improves the fertility of dairy cows following artificial insemination. Steroid hormone interactions following ovarian manipulation affect the probability of pregnancy establishment and maintenance.

## Graphical Abstract

**Graphical Abstract:**
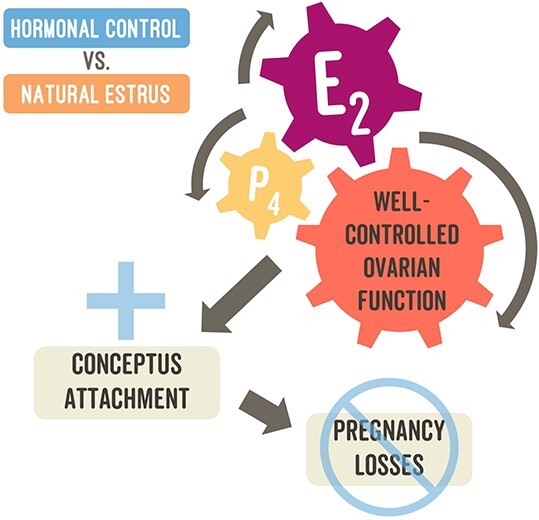

Graphical description of pre-conception dynamics of progesterone (P_4_) and estrogen (E_2_) leading to greater chances for conceptus attachment and fewer pregnancy losses in lactating dairy cows.

## Introduction

A significant gap remains unclear in the understanding of poor fertility of dairy cows. Manipulating ovarian function with exogenous hormonal intervention to limit the ovulatory follicle's antral age improves dairy cow fertility compared to cows receiving artificial insemination (AI) following estrus [1, 2]. Yet, it is unclear why these differences exist and if time of conceptus attachment may be a limiting factor.

A systematic review of fertility data published before 2016 estimated that pregnancy losses occurring from day 8 to 28 ranged between 19 and 41% [3]. This period encompasses the critical establishment of the commencing physical communication between conceptus and dam, including the period of conceptus attachment [4]. Time to conceptus attachment was highly associated with pregnancy loss [5]. Pregnancy-specific protein B (PSPB), a pregnancy-associated glycoprotein (PAG), is produced from giant trophoblast cells [6, 7] and has been successfully utilized as a pregnancy marker in the maternal circulation beginning near the time of conceptus attachment [5, 8, 9]. Middleton et al. [8] reported that most cows experienced a significant PSPB increase on day 20 or 21 of gestation from a within-cow baseline. Considering the timing of PSPB increase, it appears this event occurs at or soon after conceptus attachment. This observation corroborates previously published morphological evidence [4, 10]. Cows with a delay (≥22 days post-ovulation) in conceptus attachment had greater chances of pregnancy loss compared with most cows that had conceptus attachment on days 19 to 21 [5]. The prospective discovery of utilizing serial measurements of PSPB as early as 19 days post-AI as a marker of conceptus attachment creates an opportunity to gain a greater understanding of pregnancy losses in lactating dairy cows. This knowledge gap also involves pre- and post-conception factors that may impact pregnancy maintenance.

Pre- and post-conception factors may positively or negatively influence the outcome of AI in dairy cows. These factors include ovulatory follicle antral age or follicle diameter [11–13], estrogen (E_2_) and progesterone (P_4_) dynamics prior to ovulation (high E_2_, concomitant with low P_4_) [14–19], reproductive tract size [20–22], maintenance of the corpus luteum (CL) during maternal recognition of pregnancy [23–25], as well as conceptus-driven PSPB secretion [26–28]. It was not clear, however, if the time from AI to an initial increase in PSPB in maternal circulation could explain why cows treated with fertility programs (i.e. estrous synchronization programs that improve fertility [29]) have a greater chance for pregnancy compared with AI following natural estrus. Fertility programs generally use GnRH to manipulate follicular dynamics and PGF_2α_ to control luteal function to limit the age of the ovulatory follicle, ensure luteolysis and a specific time of ovulation, and provide the ideal association of AI with ovulation [11, 30].

The main objective of this study was to determine differences in time to conceptus attachment in lactating cows treated with the fertility program Double-Ovsynch compared with cows that were detected in estrus with automated activity monitors. Our secondary objectives were to determine the effect of treatment on pre- and post-conception factors (E_2_ and P_4_ near ovulation, uterine size, CL blood flow, and serum PSPB) and how these relate to fertility outcomes. We hypothesized that time of conceptus attachment after natural estrus AI would be delayed and result in lower PSPB concentrations compared to cows treated with Double-Ovsynch.

## Materials and methods

### Experimental units

Data were collected at Nobis Dairy Farm in St. Johns, Michigan, USA. Power analyses revealed that at least *n* = 22 pregnant lactating dairy cows per treatment were necessary to detect a 1.5-day difference between treatments in time to conceptus attachment (α = 0.05, β = 0.20, σ = 1.97 based on previously published data [5]). During the study period, *n* = 162 lactating dairy cows were due for first service and were available for inclusion in the study. Cows were considered ineligible for treatment assignment based on culling/selling decisions (*n* = 16) or health-related conditions (*n* = 1). Thus, *n* = 145 cows were assigned to treatments during six weeks, ranging from first to seventh parity. A total of *n* = 5 cows were excluded due to ovulation failure following AI. Cows were fed a total mixed ration (TMR) once daily with free access to feed and water and were confined in a free-stall barn. The TMR consisted of corn, wheat, and alfalfa silages, and corn-soybean meal-based concentrates formulated to meet or exceed nutrient recommendations for high-producing lactating dairy cows [31]. Nobis Dairy has a rolling herd average of 16,009 kg milk and 627 kg fat. The Institutional Animal Care and Use Committee at Michigan State University approved all animal handling and procedures.

### Treatments

**Figure 1:**
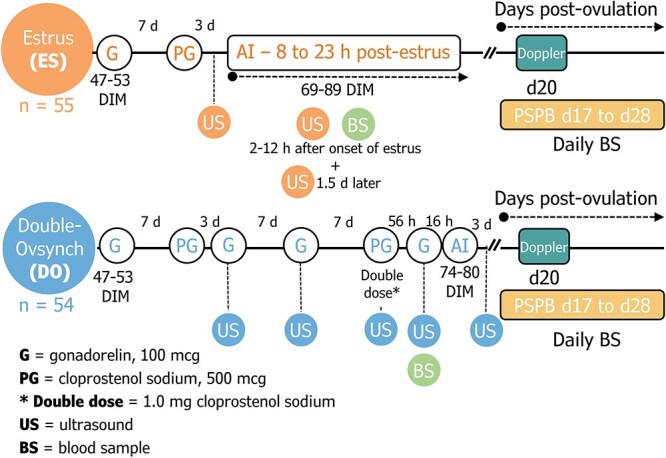
Description of treatment protocols utilized to artificially inseminate (AI) lactating Holstein cows (*n* = 109) for first service. A combination of gonadorelin (G) and cloprostenol sodium (PG) was utilized in both treatments. In the ES group, an initial treatment of G—7 days (d)—PG was given to resolve cyclicity. AI was performed between 69 and 89 days in milk (DIM), 8 to 23 h (h) after estrus onset was detected utilizing automated activity monitors. The DO cows received G and PG administrations as described below. Timed-AI was performed 16 h after the final G, between 74 and 80 DIM. Ultrasound (US) exams were performed at the day of final G (DO) or 2–12 h after estrus onset (ES), and a blood sample (BS) was collected concomitantly. Ovulation was confirmed in a second US exam, either 3 days after AI in DO cows, or 1.5 days after estrus onset in ES cows. Daily BSs were collected to measure pregnancy-specific protein B (PSPB) between Days 17 to 28 post-ovulation. On day 20, luteal function was assessed with color Doppler.

Lactation number was utilized as the blocking variable, and cows were randomly assigned to treatments in a skewed ratio (60% Estrus, 40% Double-Ovsynch) estimating that 70% of estrus cows would be detected and inseminated. Cows in the Double-Ovsynch group (DO; *n = 54*) were synchronized with GnRH (100 μg of gonadorelin; Cystorelin, Boehringer Ingelheim Animal Health) and PGF_2α_ [0.5 mg (during first Ovsynch) and 1.0 mg (during second Ovsynch) of cloprostenol sodium; Synchsure, Boehringer Ingelheim Animal Health] and received TAI (average 77 ± 0.3 days in milk, or DIM) as described in Figure 1. Cows in the estrus group (ES; *n* = 86) received GnRH (100 μg of gonadorelin) between 47 and 53 DIM, and PGF_2α_ (0.5 mg of cloprostenol sodium) 7 days later, between 54 and 60 DIM (Figure 1) to initiate cyclicity. Cows in ES were fitted with a collar and mounted with an activity and rumination monitor (Heatime Pro+ system, powered by Allflex). Percentage activity change data were updated in 2-h intervals. Alerts triggered in the DataFlow II software (Allflex Livestock Intelligence) were utilized to identify cows in estrus. Based on the software criteria, estrus onset was defined as the first time point that activity increased 35% from the 7-day activity average. Overall, 70.9% (61/86) of cows were detected in estrus and received first service, but only cows detected in estrus between 70 and 89 DIM (average 82 ± 0.7 DIM) and receiving AI between 8 and 23 h from onset of estrus were included in the analyses (*n = 55*). Artificial insemination was performed twice a day by two technicians (approximately at 07:00 and 13:00). Conventional semen from multiple sires (*n* = 4) purchased by the farm was utilized and evenly distributed across treatments.

### Ovarian dynamics and measurement of ovarian structures and uterine horns

Linear array ultrasonography (7.5 MHz, MyLabVet Delta, Esaote) was used to map ovaries and assess ovarian dynamics. All measurements were determined using built-in calipers. The first diameter measurement for each structure was horizontal, at the greatest length, and the second was perpendicular to the first measurement, at the greatest height. Ovarian dynamics in ES cows were evaluated 3 days following cloprostenol sodium administration to gather baseline information regarding cyclicity rates.

Assessment of CL volume, follicle, and uterine horn diameters before AI was performed on the day of final GnRH (DO) or 2–12 h after estrus onset (ES). Luteal volume was reported in mm^3^ and calculated with the formula volume = (4/3) π *R*^3^. *R* was the radius, or ½ the diameter in mm. The volume of cavities, if present, was calculated with the same formula and subtracted from the final luteal volume. The ovulatory follicle diameter (mm) and the uterine horn diameter (mm) were calculated as the average between its horizontal and perpendicular diameters. Average ovulatory follicle diameter was calculated only in cows with single ovulations (*n* = 42 for ES and *n* = 46 for DO). Uterine horns were measured cross-sectionally immediately cranially to the uterine bifurcation. If observed, the lumen diameter was measured and further subtracted from the final average diameter. For analyses, only the size of the horn ipsilateral to the ovulation was utilized. In cows with bilateral ovulations, the average diameter of the right and left horns was calculated. Ovulation was confirmed in all cows included in the dataset. Either 4 days after final GnRH administration for the DO treatment or 36 to 48 h after estrus onset for ES treatment (Figure 1). An additional exam was performed 20 days post-AI to assess luteal blood flow (LBF) as a morphological indicator of luteal function. Luteal blood flow was measured in individual CL utilizing color Doppler. Images were saved and processed to select the colored pixel area, as previously described [32]. Luteal blood flow was reported as the average colored pixel area from two pictures for each CL. If more than one CL was present, CL’s LBF was added.

### Blood samples for determination of E_2_, P_4,_ and PSPB concentrations

For the DO treatment, samples were collected before the final GnRH administration. This is the ideal time to sample for assessing steroid hormones because most cows will have undergone complete luteolysis and have an E_2_ active dominant follicle at this time. In the ES treatment, blood collection was performed either at 6:00 or 18:00, resulting in a minimum of 2 and a maximum of 12 h from estrus onset to sample collection. Blood samples were collected in 8.5-mL tubes from the coccygeal artery or vein into tubes coated with clot-activator, and separator gel (BD Vacutainer). The post-AI sampling regimen included daily samples between days 17 to 28 post-ovulation. Samples were allowed to clot at 4°C for 24 h before being centrifuged at 2000 × *g* for 20 min for serum separation. A 1.5-mL aliquot of serum was stored at −18°C until shipment to external laboratories for analyses (Dr. George Perry’s laboratory at Texas A&M and bioTRACKING). Samples collected before AI were assayed for P_4_ and 17β-estradiol (E_2_) with previously validated RIA assays ([33, 34], respectively). Intra- and inter-assay coefficients of variation (*CV*) for the P_4_ assays were 3.5% and 9.2%, respectively. The assay sensitivity was 0.08 ng/mL. The E_2_ assays had intra- and inter-assay CVs of 4.4% and 8.4%, respectively. Assay sensitivity was 0.5 pg/mL.

The present study design was limited by its inability to capture E_2_ and P_4_ concentrations near the LH surge in the ES treatment. Samples were collected within a 2- to 12-h range after estrus onset as detected with automated activity monitors (35% or greater increase from basal activity). The authors acknowledge that E_2_ concentrations and, consequently, the ratio of E_2_ to P_4_ could be confounded due to an extended time between estrus onset and sample collection. The minimum E_2_ concentration in the DO treatment was 1.14 pg/mL (known time of the LH surge, i.e. induced with GnRH). This value was utilized as the minimum expected E_2_ concentration threshold in ES cows (estimated time of the LH surge). A total of *n* = 10 ES cows with E_2_ concentrations below this threshold were removed from these analyses as a mitigation strategy to censor cows that had returned to basal concentrations of E_2_.

A commercial ELISA kit (bioPRYN, bioTRACKING), developed by Sasser et al*.* [35], was utilized to measure serum concentrations of PSPB in samples collected daily between days 17 and 28 post-ovulation. The lowest threshold of the assay was 0.2 ng/mL. The intra- and inter-assay CV were 4.9% and 8.3%, respectively.

### Criteria to determine the first day of continuous PSPB increase

A baseline concentration value was calculated for each cow as the average of samples collected on days 17 and 18 post-ovulation for each cow. The lowest detectable amount (0.2 ng/mL) was utilized as a baseline value when both day 17 and 18 samples were below this sensitivity cutoff. The day of a significant increase in PSPB concentrations was defined as the first day in which PSPB increased ≥12.5% from the baseline. The PSPB concentration on that day also had to be above the lowest detectable amount. Day of the initial PSPB increase is henceforth referred to as day of conceptus attachment. Two additional days of ≥12.5% increase from the previous day were utilized to confirm that PSPB was continuously increasing within the cow. The period from the initial day of PSPB increase, or conceptus attachment, in addition to the following 2 days in which PSPB continuously increased will be referred to as the conceptus attachment “confirmatory period.”

### Validation of differential steroid hormone dynamics near time of LH surge

A ratio between E_2_ and P_4_ was calculated (Ratio = E_2_ concentrations in pg/mL/P_4_ concentrations in ng/mL) to determine the interactions between these hormones. The dataset was segmented into tertiles within treatments (ES: bottom *n* = 15, mid *n* = 15, and top *n* = 14; DO: bottom, mid and top *n* = 18/tertile). These data were utilized due to the clear variability between tertiles within each treatment. Each E_2_ to P_4_ tertile differed significantly from one another (ES *P* ≤ 0.02; DO *P* < 0.01).

### Calculation of additional variables to describe embryo viability

Different daily PSPB concentration metrics were proposed to address the non-normal distribution of this variable as it relates to pregnancy status and embryonic competency. The first approach was to investigate PSPB concentrations during the confirmatory period (first, second, and third days of conceptus attachment). The second approach was to sum up the observed concentrations for the extent of the confirmatory period (3-day period), which may be referred to as cumulative PSPB concentrations.

### Pregnancy diagnoses

Cows that returned to estrus after AI were considered non-pregnant and re-inseminated, and thus not checked for pregnancy. A total of *n* = 46 cows returned to estrus after first service. From these, *n* = 6 had an indication of conceptus attachment. A total of 30/46 cows returned to estrus before or at 23 days post-AI. The remainder 16 cows returned to estrus between days 24 and 32 post-AI. The remaining cows were checked by the herd veterinarian between day 31 to 37 post-ovulation, and this is referred to as the first pregnancy diagnosis. Cows were re-confirmed between day 60 to 66 post-ovulation, and this is referred to as the second pregnancy diagnosis. Each of these pregnancy diagnoses were performed via transrectal ultrasonography.

Cows were also grouped across treatments into three categories of pregnancy status: cows with no conceptus attachment (No-CA), cows with conceptus attachment that maintained pregnancy until the second pregnancy diagnosis (Maintained), and cows with conceptus attachment that lost pregnancy at any timepoint up to the second pregnancy diagnosis (Lost). Pre- and post-conception factors with possible detrimental effects on pregnancy establishment and survival were investigated between treatment and between pregnancy status.

### Statistical analyses

All statistical analyses were performed using SAS (version 9.4, SAS Institute Inc.). The level of significance was set at *P* ≤ 0.05. A tendency was described as a *P-*value ranging from >0.05 to ≤0.10. Multiple comparisons were adjusted with the Tukey–Kramer test for multiplicity. Descriptive statistics utilized features of PROC FREQ and PROC MEANS. All continuous variables were reported as arithmetic means ± SEM.

Analysis of residuals from continuous variables was utilized to assess normality with PROC UNIVARIATE and SGPLOT. Shapiro–Wilk was utilized as an objective normality test. A Box-Cox transformation analysis was employed utilizing PROC TRANSREG on variables that presented a non-normal distribution to establish the value of the λ-exponent. The following variables were transformed with the recommended λ exponents in parenthesis: follicle diameter (−0.85), total CL volume prior to AI (0.3), horn diameter (log), E_2_ concentrations (0.2), P_4_ concentrations (0.3), ratio E_2_ to P_4_ (0.25), total pixels at day 20 post-AI (0.6), PSPB concentration (−0.15), and sum PSPB concentrations during confirmatory period (log). Further analyses were performed with the transformed variable. Figures were built in Excel with untransformed variables to aid data interpretation.

Analyses were performed utilizing PROC MIXED. All statistical models included the fixed effects of treatment and parity (primiparous *n* = 36, multiparous *n* = 73). When applicable, pregnancy status and double ovulation were also included as fixed effects. Lactation (first to seventh) was included as the block variable in the random statement. The classification of conceptus attachment day was included as a random effect (days 19, 20, 21, and ≥ 22) when analyzing the sum of PSPB concentration during the confirmatory period. All analyses of E_2_ to P_4_ ratio were performed separately within ES and DO treatments and no treatment comparisons were inferred.

Repeated measurements were analyzed with PROC MIXED and included time in the REPEATED statement. Models included time, treatment, and parity as fixed effects, in addition to pregnancy status when applicable. The random statement included lactation, day of conceptus attachment classification, and ID nested within treatment. ID nested within treatment was also specified in the subject option. A first-order autoregressive [AR(1)] covariance structure was utilized.

Predicted probabilities were estimated with PROC LOGISTIC. Wald chi-square was used to test the association between pre- and post-conception continuous variables and outcome of interest (conceptus attachment or pregnancy loss). The 95% confidence interval for the predicted probabilities was also estimated with PROC LOGISTIC. The linear relationship between two continuous variables was estimated with PROC REG utilizing a univariate model and tested with a two-tailed *t*-test. PROC CORR was utilized to estimate Pearson correlation coefficients between two continuous variables.

## Results

### Effect of treatment on days to conceptus attachment

**Figure 2:**
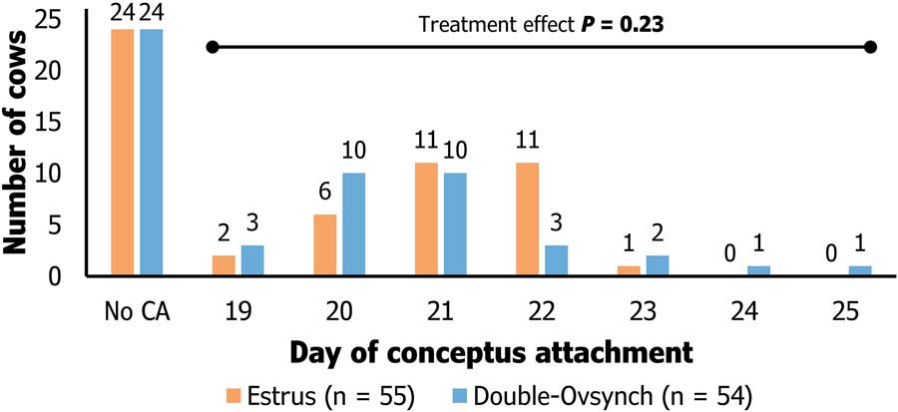
The effect of treatment on the distribution of day of conceptus attachment in lactating Holstein cows (*n* = 109). Treatments consisted of AI following either estrus detection (ES, *n* = 55) or the fertility program Double-Ovsynch (DO, *n* = 54).

There was no difference in the distribution of days to conceptus attachment between treatments (Figure 2; *P* = 0.23). The average days to conceptus attachment did not differ between ES vs. DO treatments (21.1 ± 0.2 vs. 20.9 ± 0.3; *P* = 0.19) or between primiparous and multiparous cows (21.2 ± 0.2 vs. 20.8 ± 0.2; *P* = 0.13). Conceptus attachment occurred in 31/55 ES and 30/54 DO cows. There was a tendency for greater pregnancy losses following ES compared with DO (7/31 vs. 1/30; *P* = 0.06) between the time of conceptus attachment and the first pregnancy diagnosis. From day of conceptus attachment until the second pregnancy diagnosis between day 60 to 66 post-AI, 8/31 ES and 4/30 DO cows lost pregnancy. Average days from AI to conceptus attachment in cows that maintained vs. lost pregnancy from conceptus attachment to pregnancy diagnosis with ultrasound on day 60 to 66 post-AI was 21.1 ± 0.2 vs. 21.1 ± 0.5 for ES and 20.8 ± 0.3 vs. 21.8 ± 1.1 for DO (*P* ≥ 0.35).

### Effect of treatment on serum concentrations of PSPB during the first 3 days of conceptus attachment

**Figure 3:**
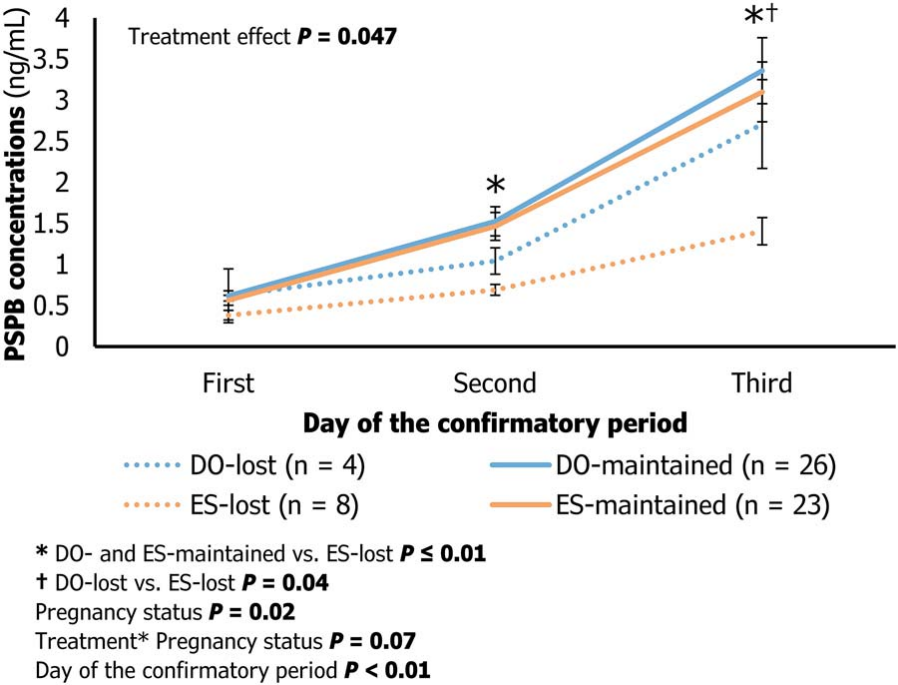
The effect of treatment (Trt; Estrus detection, ES, or Double-Ovsynch, DO) on concentrations of pregnancy-specific protein B (PSPB; ng/mL) during the confirmatory period. The confirmatory period consisted of the first day of significant PSPB increase in the maternal circulation (“first” or day of conceptus attachment), in addition to two more days in which PSPB was continuously increasing (“second” and “third”). The pregnancy status “maintained” included cows with conceptus attachment that sustained pregnancy to 60–66 days after AI.

Concentrations of PSPB during the confirmatory period (first 3 days of conceptus attachment) are described in Figure 3. There were no differences (*P* = 1.0) in concentrations of PSPB during the confirmatory period between treatments in cows that maintained pregnancy. Cows in the ES treatment that lost pregnancy had lower PSPB concentrations on the second and third day of the confirmatory period in comparison with ES and DO cows that maintained pregnancy (*P* ≤ 0.04). On the third day, there were greater PSPB concentrations in DO compared with ES (*P* = 0.04) in cows that lost pregnancy. Parity had no effect on PSPB concentrations during the confirmatory period (*P* = 0.53).

### Effect of treatment on pre-conception factors

Follicle diameter, uterine horn diameter, serum E_2_, and P_4_ concentrations, and the E_2_ to P_4_ ratios were measured near the time of the LH surge in each treatment (Figure 1). Follicle diameter was greater for ES in comparison with DO (18.4 ± 0.5 vs. 14.9 ± 0.3 mm; *P* < 0.01). Horn diameter tended to be greater in ES cows in comparison with DO cows (22.5 ± 0.6 vs. 21.8 ± 0.6 mm; *P* = 0.08). Concentrations of E_2_ (4.0 ± 0.4 vs. 3.9 ± 0.3 pg/mL; *P =* 0.78) and P_4_ (0.49 ± 0.04 vs. 0.60 ± 0.04 ng/mL; *P =* 0.78) did not differ between ES and DO. No effect of parity was observed for any of the analyzed pre-conception variables (*P* ≥ 0.18).

### Validation of the E_2_ to P_4_ ratio as a descriptor of steroid hormone dynamics

**Figure 4:**
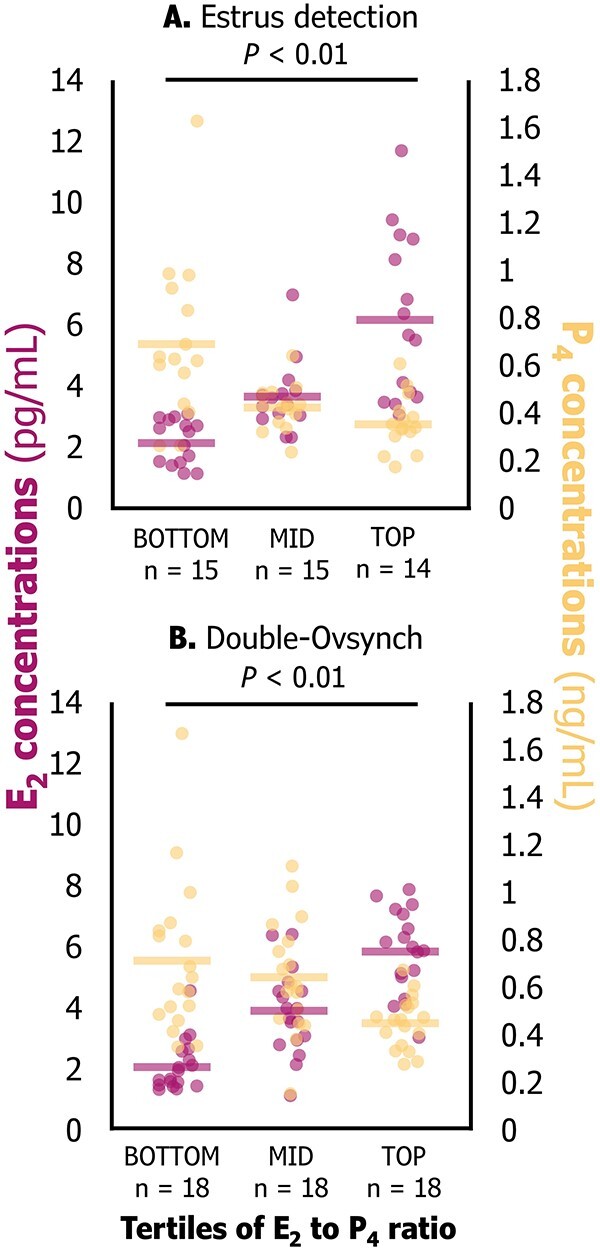
Description of the relationship between circulating concentrations of 17β-estradiol (E_2_) and progesterone (P_4_) within assigned E_2_ to P_4_ ratio tertiles (bottom, mid and top) and treatments (A. Estrus detection or B. Double-Ovsynch). Concentrations of E_2_ and P_4_ were measured in serum, from samples collected at the day of the final gonadorelin (Double-Ovsynch) or 2–12 h following estrus onset (Estrus detection). Each circle represents an observation, and the lines denote the average for E_2_ (in pink) or P_4_ (in gold) concentrations within tertile and treatment. Average E_2_ and P_4_ concentrations differed between tertiles (*P* < 0.01) for comparisons performed within treatments.

There was a wide range in the E_2_ to P_4_ ratio near the time of the LH surge within both ES and DO treatments. In the ES treatment, the E_2_ to P_4_ ratio averaged 3.7 for the bottom tertile (range 0.7–5.8), 8.5 for the mid tertile (range 6.9–10.9), and 18.6 for the top tertile (range 11.7 to 35.3). In the DO treatment, the E_2_ to P_4_ averaged 3.3 for the bottom tertile (range 1.2–4.6), 6.1 for the mid tertile (range 5.1–7.4), and 13.7 for the top tertile (range 7.5–23.8). Additionally, and as expected, concentrations of E_2_ increased and P_4_ decreased from the lowest to highest tertiles of E_2_ to P_4_ ratios (Figure 4). Whether ovulation induction was manipulated through PGF_2α_ and GnRH or the LH surge occurred endogenously, the distribution of cows within each E_2_ to P_4_ ratio tertile and the patterns of E_2_ and P_4_ within those ratios remained consistent. A total of n = 10 DO cows presented a ≥35% increase in activity before the administration of GnRH. These cows could have had an endogenous LH surge prior to GnRH administration. These cows had greater E_2_ to P_4_ ratio in comparison with cows that had no estrus expression before the final GnRH of Double-Ovsynch (10.9 ± 2.3 vs. 7.0 ± 0.7; *P* = 0.05). No differences in time of conceptus attachment were observed between these cows and cows that had no estrus expression before GnRH administration (21.1 ± 0.3 vs. 20.3 ± 0.3; *P* = 0.25).

**Figure 5:**
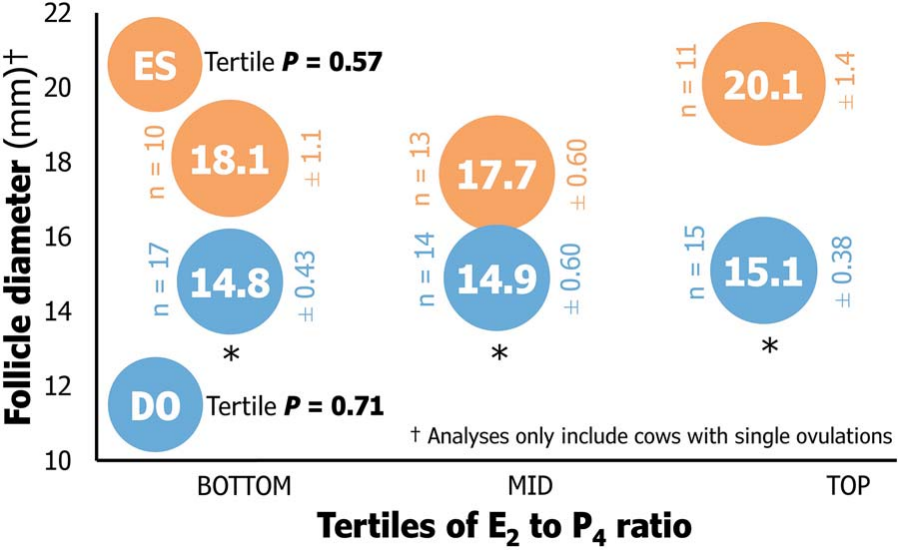
The relationship between ovulatory follicle diameter (mm; in cows with confirmed single ovulations) and tertiles of the ratio of 17β-estradiol (E_2_) to progesterone (P_4_) with each treatment (Estrus detection or ES, and Double-Ovsynch or DO). Follicle diameter was measured with ultrasound at the day of the final gonadorelin (DO) or 2–12 h following estrus onset (ES). Follicle diameter did not differ between tertiles in analyses performed within treatments (*P* ≥ 0.56). The symbol * denotes *P* ≤ 0.04 for comparisons of DO vs. ES within tertiles.

There was no linear relationship between luteal volume measured on the day of estrus or the final GnRH, and the E_2_ to P_4_ ratio (*P* = 0.66). There was an overall positive linear relationship between ovulatory follicle diameter and the E_2_ to P_4_ ratio (*P* = 0.01). Ovulatory follicle diameter did not differ between tertiles within ES and DO treatments (Figure 5). However, ES had a greater average ovulatory follicle diameter in comparison with DO in each tertile of E_2_ to P_4_ ratios (*P* ≤ 0.04).

### Effect of treatment on post-conception factors

Treatment and parity effects were determined for the post-conception factors LBF and cumulative PSPB concentrations during the confirmatory period. There were no differences in LBF measured as colored pixels at day 20 post-AI between treatments (9,831.2 ± 921.9 for ES vs. 9,547.2 ± 820.2 for DO; *P* = 0.76). Logistic regression analyses indicated that LBF was predictive of conceptus attachment in both treatments (*P* < 0.01). Finally, no treatment effects were observed for the sum of PSPB concentrations during the confirmatory period (4.4 ± 0.5 vs. 5.3 ± 0.6 ng/mL for ES vs. DO, respectively; *P* = 0.14). Cumulative PSPB during the confirmatory period was associated with the predicted probability of pregnancy loss in ES but not DO (*P* = 0.02 vs. *P* = 0.66). No significant parity effects were observed for any of the analyzed post-conception variables (*P* ≥ 0.16).

### Follicular function markers as predictors of conceptus attachment

**Figure 6:**
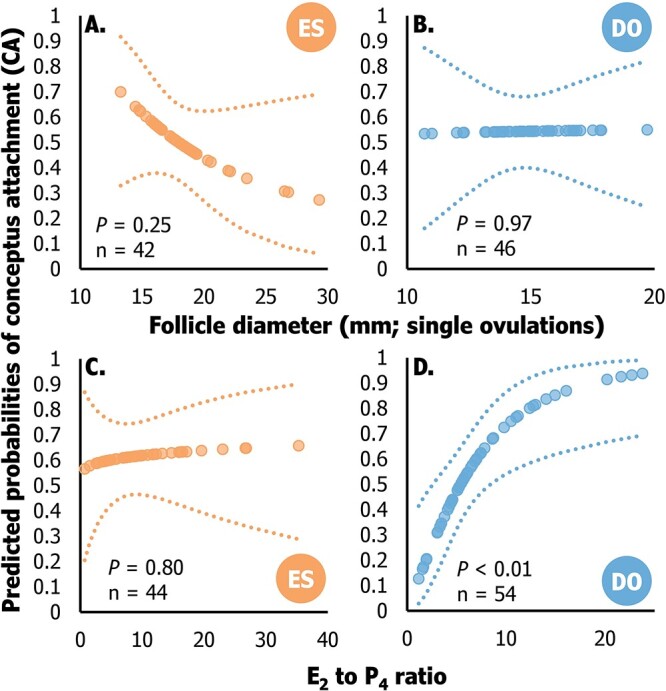
The predicted probabilities of conceptus attachment based on ovulatory follicle diameter (panels A and B) and the ratio of 17β-estradiol (E_2_) to progesterone (P_4_; panels C and D) within treatment (Estrus detection—ES, or Double-Ovsynch—DO) in lactating dairy cows. All variables were measured on the day of the final gonadorelin (DO) or 2 to 12 h after estrus onset (ES). The dotted lines represent the 95% confidence limit (upper and lower) for the predicted probabilities of conceptus attachment.

**Figure 7:**
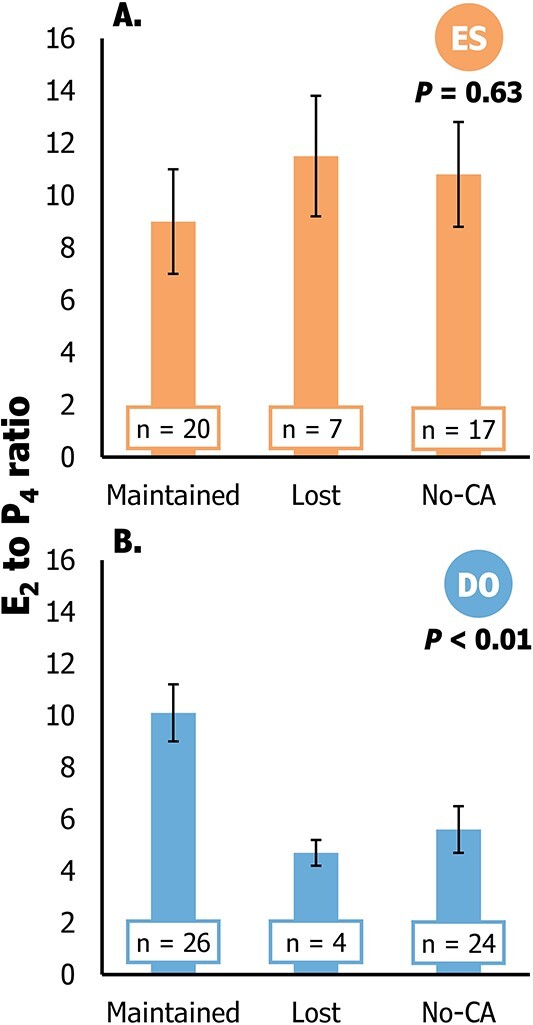
A description of the 17 β-estradiol (E_2_) to progesterone (P_4_) ratios in cows with and without conceptus attachment and in cows that had pregnancy loss following conceptus attachment within each treatment (Estrus detection or ES, and Double-Ovsynch or DO). Pregnancy statuses were determined utilizing the day of conceptus attachment (CA) as the initial baseline. The “Maintained” status included cows with conceptus attachment that sustained pregnancies up to the second pregnancy diagnosis (Days 60 to 66 post-artificial insemination—AI). The “Lost” status included cows that had conceptus attachment and lost pregnancy at any time point up to the second pregnancy diagnosis. And cows in the “No-CA” status were cows with undetected PSPS increase in maternal circulation.

Follicle diameter did not emerge as a significant predictor of conceptus attachment for either treatment (Figure 6). However, the E_2_ to P_4_ ratio exhibited a positive association with the probability of conceptus attachment in DO but not in ES (Figure 6). In the DO treatment, the E_2_ to P_4_ ratio had a linear (*P* < 0.01) association with predictions of conceptus attachment. Additionally, DO cows in the bottom, middle and top tertiles of E_2_ to P_4_ ratio had 33.3 (6/18), 55.5 (10/18) and 77.7% (14/18) conceptus attachment. Distinct variations between treatments were evident in the average E_2_ to P_4_ ratio among cows experiencing conceptus attachment and maintaining pregnancy (maintained), those with conceptus attachment followed by pregnancy loss (Lost), and those without conceptus attachment (No-CA; Figure 7). Cows in the DO group with conceptus attachment that maintained pregnancy to 60–66 days post-AI displayed a greater average E_2_ to P_4_ ratio compared to cows with conceptus attachment and subsequent pregnancy loss, as well as those without conceptus attachment (*P* ≤ 0.05).

### Associations of conceptus attachment outcome and pre- and post-conception factors

The impact of overall pregnancy status on pre- and post-conception limiting factors is elucidated in Table 1 (data from ES and DO treatments combined). The E_2_ to P_4_ ratio tended to be differently affected by the pregnancy status. Cows that maintained pregnancy tended to have a greater E_2_ to P_4_ ratio in comparison with cows that had no conceptus attachment (*P* = 0.07). Conversely, E_2_ to P_4_ ratios were not different in cows with pregnancy loss compared to both cows that maintained pregnancy or that had no conceptus attachment (*P* ≥ 0.35). Luteal blood flow was not different in cows with pregnancy loss compared to cows that maintained pregnancy (*P* = 0.35), but both groups with conceptus attachment had greater LBF compared to cows with no conceptus attachment (*P* < 0.01). The cumulative PSPB concentrations during the confirmatory period were greater for cows that maintained pregnancy compared to cows with pregnancy loss following conceptus attachment (*P* = 0.01).

**Table 1:** Relationship of pregnancy status with pre- and post-conception factors

	Maintained *n* = 49	Lost *n* = 12	No-CA *n* = 48	*P*-value
Pre-conception factors				
Follicle diameter (mm)	16.0 ± 0.3	16.5 ± 0.7	17.1 ± 0.6	0.89
Uterine horn diameter (mm)	21.2 ± 0.6	23.3 ± 1.4	22.9 ± 0.6	0.12
E_2_ concentrations (pg/mL)	4.0 ± 0.3	3.4 ± 0.5	3.4 ± 0.4	0.55
P_4_ concentrations (ng/mL)	0.46 ± 0.02	0.45 ± 0.06	0.64 ± 0.05	0.61
E_2_ to P_4_ ratio	9.3 ± 0.8	8.4 ± 1.6	7.4 ± 1.1	0.09
Post-conception factors				
LBF (×1000 pixels)	13.24 ± 0.78^a^	10.96 ± 1.55^a^	5.78 ± 0.76^b^	<0.01
Time to CA (days)	20.9 ± 0.2	21.3 ± 0.5	–	0.17
Cumulative PSPB (ng/mL)	5.3 ± 0.4^a^	3.1 ± 0.4^b^	–	0.03

Pregnancy statuses were determined utilizing the day of significant PSPB increase, or day of CA as the initial baseline. The “maintained” status included cows with CA that sustained pregnancies up to the second pregnancy diagnosis (Days 60 to 66 post-artificial insemination—AI). The “Lost” status included cows that had CA and lost pregnancy at any time point up to the second pregnancy diagnosis. And cows in the “No-CA” status were cows with undetected PSPB increase in maternal circulation. ^a, b^Different letter superscripts denote a *P* ≤ 0.03.

### Relationships between cow- and conceptus-related factors.

Ovulatory follicle (*P* ≥ 0.10) and uterine (*P* ≥ 0.79) diameters were not correlated with days to conceptus attachment or cumulative PSPB during the confirmatory period. However, horn diameter had a significant negative correlation with cumulative concentrations of PSPB in ES but not DO treatments (ES *r* = −0.36, *P* = 0.049; DO *r* = −0.03, *P* = 0.87). Concentrations of E_2_ and P_4_ near time of the LH surge were not correlated with the conceptus-related investigated factors (*P* ≥ 0.13 and *P* ≥ 0.14, respectively). There was no relationship observed between E_2_ to P_4_ ratio and days to conceptus attachment (*P* ≥ 0.26), but a greater E_2_ to P_4_ ratio tended to have a negative correlation with the cumulative PSPB concentrations in ES but not DO-treated cows (ES *r* = −0.32, *P* = 0.08; DO *r* = 0.1, *P* = 0.60). There was no significant correlation between LBF at day 20 post-AI and days to conceptus attachment, or cumulative PSPB concentrations over the confirmatory period (*P* ≥ 0.15).

## Discussion

The objective of this study was to gain a greater understanding of mechanisms involved in greater pregnancies per AI (P/AI) attained with fertility programs, such as Double-Ovsynch [1], compared to AI following the detection of estrus. Our laboratory developed a model for estimating the occurrence and time of conceptus attachment using a conservative calculation of daily increases in maternal circulating concentrations of PSPB, approximately around day 20 post-AI [8]. This innovation enabled us to investigate the developmental aspects of embryonic growth two weeks earlier than the conventional day ~35 pregnancy diagnosis. A delay in the timing of conceptus attachment clearly indicates an elevated risk of pregnancy loss [5]. Such losses would go unnoticed at the standard 35 days post-AI pregnancy evaluation.

Contrary to our hypotheses, there were no differences between treatments in time to conceptus attachment or in cumulative serum concentrations of PSPB during the first 3 days of conceptus attachment (Figures 2 and 3). In a previous study, more than 85% of cows with conceptus attachment on days 20 and 21 post-AI (estimated based on initial PSPB increase) sustained their pregnancy [5]. The ability of a conceptus to secrete products into the maternal circulation in a timely manner is suggestive of proper development and successful activation of molecular pathways involved with conceptus attachment. Adhesion is the initial process that leads to stable cell-to-cell interaction, conceptus attachment, and placentation [4, 10, 36]. This process is mediated through several molecules, but integrins and cadherins were largely characterized during the peri-attachment period in ruminants [37–39]. The extended period to detect PSPB in the maternal circulation (conceptus attachment ≥22 days; 19/61 cows) in the current study could be associated with malfunction or delay in initial steps of conceptus attachment and giant trophoblast cell differentiation [40]. The phenotype of delayed increase in PSPB was less prevalent in previously sampled populations [5, 8]. Thus, it becomes highly likely that subtle differences in average days to conceptus attachment are uncaptured in small sample sizes even though power analyses were performed using these previous data to determine sample size.

Cows in the ES group that lost pregnancy had diminished concentrations of PSPB following conceptus attachment, a phenotype not observed in DO cows that also lost pregnancy in this study. Santos et al*.* [5] reported a clear reduction in PSPB secretion during the first week of conceptus attachment in cows with early pregnancy loss. The occurrence of compromised PSPB secretion, concurrent with unsuccessful pregnancies, may be associated with the premature pregnancy losses observed in 7/8 cows in the ES treatment (before the first pregnancy diagnosis). Lower concentrations of PSPB at day 24 post-AI were an early predictor of pregnancy loss [26, 28]. The present study is the first report of a trend for greater pregnancy loss as a plausible explanation for reduced P/AI following estrus detection. Studies with greater numbers of cows are needed to evaluate the effect of ES and DO on the proportion of early pregnancy losses occurring past conceptus attachment.

The mechanism(s) of reduced PSPB in serum of cows that lost pregnancies and the variation in timing to conceptus attachment is not clear and was not an objective of this study. Is this due to a slowed development of the conceptus or a flaw in the ability of giant trophoblast cells of a healthy and normal developing conceptus to produce and transfer adequate quantities of PSPB into maternal circulation? Thus, are adequate quantities of PSPB needed for conceptus attachment or are the lower concentrations of the PSPB a consequence of a retarded conceptus? The role of PSPB in pregnancy establishment and survival has not been fully characterized. *In vitro* evidence demonstrated that uterine transcriptome can be altered with PAG exposure. Genes associated with tissue remodeling were upregulated in both pregnant and non-pregnant uterine explants upon treatment with PAG [41]. The same authors proposed a maternal-conceptus interface localized action. PSPB could act as regulators of tissue remodeling that accompanies conceptus attachment and subsequent placentation. The presence of PAG-positive trinucleate trophoblast cells incorporated into the uterine endometrium was reported as early as day 20 [42], alongside firm attachment of the trophectoderm to the endometrial lining between days 20 and 21, ipsilateral to the CL [10]. Below average and/or delayed PSPB exposure to the maternal interface during this critical time may be a limiting factor to sustaining pregnancy. Pregnancies presenting these types of PSPB secretion profiles were more likely to be terminated before a day 34 pregnancy diagnosis [5]. There was about 1 ng/mL less PSPB being secreted during the first 3 days following conceptus attachment in the ES in comparison with DO treatment. This decreased exposure to PSPB could be deleterious to proper pregnancy survival. In this study, the cumulative PSPB concentrations during the confirmatory period was predictive of reduced pregnancy survival in ES but not in DO. This is likely due to the trend for more losses following conceptus attachment in the ES group. Overall, conceptuses of failed pregnancies had ~2 ng/mL less PSPB over the confirmatory period in comparison with conceptuses of sustained pregnancies. Thus, this metric is a valid marker of not only presence of a conceptus but also embryonic viability of high-risk pregnancies.

In the current study, we measured various fundamental pre- and post-conception factors that can impact fertility of lactating dairy cows [15, 18–21, 24, 27, 28, 43]. Follicle diameter prior to ovulation, a key determinant of fertility [11, 44], was greater in the ES treatment compared with cows in the DO treatment. A limitation of the present study was the inability to determine the time of follicular wave emergence for the ovulatory follicle in ES cows. Based on the comparison of ultrasound scans performed 3 days following the pre-synchronization to induce cyclicity, 2/55 of cows in the ES group may have had a persistent follicle at time of estrus. These two cows, of course, ovulated these persistent follicles and were kept in the study based on our inclusion criteria. The efficiency of Double-Ovsynch in controlling the onset of follicular development, luteolysis (day 7 of development), and ovulation (9.5 days of development) results in consistent ovulation of follicles around ~16 mm. In this study, ES cows ovulated follicles on average 3.5 mm larger in comparison with DO. Considering a daily growth of 1.2 mm [45], this finding could be interpreted as the ovulation of follicles that were almost 3 days older in the ES group. This difference in ovulatory follicle diameter could be the result of more frequent LH pulses that likely lead to greater aged and prematurely matured oocytes compared to Double-Ovsynch [46]. Consequently, this suboptimal physiological state leads to decreased embryo quality following estrus detection [47]. Yet, concentrations of E_2_, which were shown to impact fertility [48, 49], were not different between treatments. Also, there was no association of the ovulatory follicle diameter and PAG concentrations between days 24 to 60 post-AI in suckled beef cows [43]. In the current study, the E_2_ secretory capacity of the pre-ovulatory follicle tended to be positively correlated with its diameter (*r* = 0.35, *P* < 0.01, data not shown).

The work presented herein proposed a combined metric to assess steroid hormone dynamics around the LH surge in both treatments. This metric consisted of a ratio between E_2_ and P_4_ concentrations (ratio E_2_ to P_4_) at a critical time following luteolysis and near the time of the LH surge. There were clear differences in the associations in the E_2_ to P_4_ ratio and conceptus attachment within each treatment. In the DO group, the greater the E_2_ to P_4_ ratio the greater chance for conceptus attachment. This was not the case in the ES group. There could be two explanations to this: (1) Double-Ovsynch controlled ovarian development in a way that limited the diameter of the ovulatory follicle to ~15 mm compared to cows detected in estrus that had follicles ~18 mm in diameter. This phenotypic difference in follicle development may have influenced these various physiological measurements. (2) The sampling period of 2 to 12 h following the onset of estrus for the ES group may not have allowed for accurate determination of E_2_ concentrations considering that E_2_ decreases following the LH surge. The LH surge appears to be highly associated with the onset of estrus in this study utilizing automated activity monitors. All cows in this study had ovulation within 36 h after estrus onset. Time from the LH surge to ovulation ranges from 24 to 32 h [50]. Thus, timing of sampling for E_2_ was likely in a period of declining concentrations.

An optimal time for measuring E_2_ and P_4_ would be near the time of the LH surge. At this stage of the estrous cycle, an inverse relationship between E_2_ and P_4_ should be ideal. Dynamic secretion and timely exposure to steroid hormones before ovulation are part of mechanistic priming of the endometrium [18] and were attributed to high fertility following both fertility programs [46] and estrus detection [51]. Complete luteolysis and reduction of P_4_ concentrations occurring synergistically with final follicular development and peaking E_2_ secretion [52] are events that set what could be referred to as the “uterine clock” [18]. Four clusters of genes were upregulated in E_2_-exposed endometrium in a model that mimicked estrous cycle changes in P_4_. These genes were associated with embryonic development, cell division/differentiation/adhesion/migration, gastrulation, organogenesis, angiogenesis, invasive growth, epithelial to mesenchymal transition, and migration, all essential to pregnancy establishment, and maintenance [18]. Motta et al*.* [53] reported a greater rate of augmentation in the endometrial area during proestrus in cows with the highest E_2_ and lowest P_4_ concentrations. This classification could be considered equivalent to the top tertiles of E_2_ to P_4_ ratio reported herein. An increased endometrial thickness the day before AI was associated with greater fertility in lactating dairy cows [54]. Thus, suboptimal and/or non-timed exposure to steroid hormones could offset the uterine clock and result in an inappropriate environment for pregnancy in cows. As previously stated cows in the top E_2_ to P_4_ ratio tertile had the highest E_2_ and lowest P_4_ concentrations. The opposite was observed on the bottom tertile of the ratio distribution. This metric described extreme scenarios of optimal and suboptimal steroid hormone interaction that were accordingly associated with fertility in the DO treatment. Cows without conceptus attachment had a lower mean E_2_ to P_4_ ratio in comparison with cows that had conceptus attachment and maintained pregnancy, regardless of treatment. This suggests that an imbalanced interaction of E_2_ and P_4_ around AI may be more limiting to conceptus attachment than pregnancy survival following conceptus attachment.

Color Doppler ultrasonography was utilized to assess luteal function and predict non-pregnant cows as early as day 20 post-AI [23, 24, 55]. In the present study, LBF at day 20 post-AI was predictive of pregnancy establishment in both treatments. Luteal rescue during maternal recognition of pregnancy is mediated through interferon-tau secretion in trophectoderm cells [25, 56, 57]. On day 21 of gestation, no differences in mRNA expression of interferon-stimulated gene-15 were reported between cows that either maintained or lost pregnancy by day 40 of gestation [58]. However, with greater number of cows, both Wijma et al. [59] and Domingues et al. [60] indicated greater interferon-stimulated gene-15 mRNA expression on days 22 and 19 of gestation, respectively, in cows that maintained pregnancy. In the present study, LBF at day 20 post-AI was not predictive of pregnancy loss. It appears that luteal rescue mechanisms were in place regardless of whether conceptuses were maintained or lost after conceptus attachment. Alternative explanations for pregnancy loss not associated with conceptus developmental issues or LBF may include pathways related to post-attachment processes, such as the epithelial-mesenchymal transition [38], tissue remodeling [10, 61], and angiogenesis leading up to placentation [62].

In summary, time to conceptus attachment did not differ between treatments. The small numbers of cows in this study did not lead to an explanation of why differences in fertility occur in cows receiving AI following estrus vs. the fertility program Double-Ovsynch. However, concentrations of PSPB during the first 3 days following conceptus attachment was a powerful predictor of pregnancy loss. This was evident in ES but not in DO. The ratio of E_2_ to P_4_ was positively associated with probability of conceptus attachment in DO treatment, but not in ES. In the DO treatment, cows that maintained pregnancy had greater E_2_ to P_4_ ratios in comparison with cows that were non-pregnant, or that lost pregnancy. This implies that maintaining adequate steroid hormone dynamics before AI positively influences the probability of both establishing and sustaining pregnancy in fertility programs. Residual P_4_ following luteolysis appears to hinder pregnancy establishment [14, 15], as does insufficient estradiol [19, 63]. Thus, it appears that fertility of lactating dairy cows is at least partially dependent upon ensuring complete luteolysis in addition to a well-controlled antral-aged pre-ovulatory follicle with the greatest steroidogenic capacity to allow for the greatest E_2_.

## Data Availability

Data is available upon email request to corresponding author (pursleyr@msu.edu).
